# Profile of *Myracrodruon urundeuva* Volatile Compounds Ease of Extraction and Biodegradability and In Silico Evaluation of Their Interactions with COX-1 and iNOS

**DOI:** 10.3390/molecules27051633

**Published:** 2022-03-01

**Authors:** Yuri G. Figueiredo, Eduardo A. Corrêa, Afonso H. de Oliveira Junior, Ana C. d. C. Mazzinghy, Henrique d. O. P. Mendonça, Yan J. G. Lobo, Yesenia M. García, Marcelo A. d. S. Gouvêia, Ana C. C. F. F. de Paula, Rodinei Augusti, Luisa D. C. B. Reina, Carlos H. da Silveira, Leonardo H. F. de Lima, Júlio O. F. Melo

**Affiliations:** 1 Departamento de Ciências Exatas e Biológicas, Campus Sete Lagoas, Universidade Federal de São João Del-Rei, Sete Lagoas 35700-000, MG, Brazil; yuri.gfigueiredo@hotmail.com (Y.G.F.); afonsohoj@gmail.com (A.H.d.O.J.); anamazzinghy@yahoo.com.br (A.C.d.C.M.); hp.quimico@hotmail.com (H.d.O.P.M.); jenny_thesiba@hotmail.com (Y.M.G.); leofrancalima@ufsj.edu.br (L.H.F.d.L.); 2 Campus Dona Lindu, Universidade Federal de São João Del-Rei, Divinópolis 35501-296, MG, Brazil; eduardo@epamig.br (E.A.C.); yanjeronimo88@gmail.com (Y.J.G.L.); 3 Empresa de Pesquisa Agropecuária de Minas Gerais, Unidade EPAMIG ITAC, Pitangui 35650-000, MG, Brazil; 4 Departamento de Ciências Agrárias, Instituto Federal de Educação, Ciência e Tecnologia de Minas Gerais, Campus Bambuí, Bambuí 38900-000, MG, Brazil; marcelogouveiatc@gmail.com (M.A.d.S.G.); ana.paula@ifmg.edu.br (A.C.C.F.F.d.P.); 5 Departamento de Química, Campus Pampulha, Universidade Federal de Minas Gerais, Belo Horizonte 35702-031, MG, Brazil; augusti.rodinei@gmail.com; 6 Instituto de Ciências Naturais, Humanas e Sociais, Universidade Federal de Minas Gerais, Belo Horizonte 35702-031, MG, Brazil; luisabarrett@gmail.com; 7 Instituto de Ciências Tecnológicas, Campus Itabira, Universidade Federal de Itajubá, Itabira 35903-087, MG, Brazil; carlos.silveira@gmail.com

**Keywords:** cerrado, Anacardiaceae, aroeira, biological properties, virtual screening, chemoinformatics

## Abstract

*Myracrodruon urundeuva* Fr. Allem. (Anacardiaceae) is a tree popularly known as the “aroeira-do-sertão”, native to the caatinga and cerrado biomes, with a natural dispersion ranging from the Northeast, Midwest, to Southeast Brazil. Its wood is highly valued and overexploited, due to its characteristics such as durability and resistance to decaying. The diversity of chemical constituents in aroeira seed has shown biological properties against microorganisms and helminths. As such, this work aimed to identify the profile of volatile compounds present in aroeira seeds. Headspace solid phase microextraction was employed (HS-SPME) using semi-polar polydimethylsiloxane-divinylbenzene fiber (PDMS/DVB) for the extraction of VOCs. 22 volatile organic compounds were identified: nine monoterpenes and eight sesquiterpenes, in addition to six compounds belonging to different chemical classes such as fatty acids, terpenoids, salicylates and others. Those that stood out were p-mentha-1,4, 4(8)-diene, 3-carene (found in all samples), caryophyllene and cis-geranylacetone. A virtual docking analysis suggested that around 65% of the VOCs molar content from the aroeiras seeds present moderate a strong ability to bind to cyclooxygenase I (COX-I) active site, oxide nitric synthase (iNOS) active site (iNOSas) or to iNOS cofactor site (iNOScs), corroborating an anti-inflamatory potential. A pharmacophoric descriptor analysis allowed to infer the more determinant characteristics of these compounds’ conferring affinity to each site. Taken together, our results illustrate the high applicability for the integrated use of SPME, in silico virtual screening and chemoinformatics tools at the profiling of the biotechnological and pharmaceutical potential of natural sources.

## 1. Introduction

Aroeira (*Myracrodruon urundeuva*), commonly known in Brazil as “aroeira do sertão” is a dioecious species. Its fruits have an oval shape, and a firm calyx, considered fruit-seed [1,2]. This species comes from cerrado and semiarid regions [3] and can be found in Brazil from the Northeast to the South of the country, presenting significant economic value [4,5,6].

Aroeira has been widely studied due to its therapeutic properties, with emphasis on anti-inflammatory, antioxidant, antimicrobial and healing activities. Its peel, leaves and fruits are used [7]. A set of recent studies have also pointed to the medicinal properties of the whole plant, particularly the extracts and essential oils from the seeds [8,9,10]. The complex mixture of aroeira seed organic compounds has also shown anthelmintic and antimicrobial activity [11].

Certain bioactive compounds present in the aroeira species, such as flavonoids, terpenes and tannins, have already been probed to exert anti-inflammatory effects with great proficiency, for instance in selectively inhibiting the phospholipase A2, an enzyme that has pro-inflammatory activity [12,13,14]. In the literature, there are several reports that the use of aroeira’s hydroalcoholic extract improves healing of several variations of inflammatory conditions and therefore, provides better healing aspects [15].

In particular, essential oils (EOs) are mainly made up of terpenes, terpenoids and their derivatives; for which evolution has selected a myriad of chemical groups and cross interactions with an equal number of biological targets in plants and animals; presenting these targets’ varied functions [16]. Allied to this, the EOs molecular constituents are usually small and lipophilic plant metabolites, with a natural ability to overcome biological barriers, in order to presenting ease of extraction and biodegradability [17,18]. This set of attributes means these plant extracts have been used in natural medicine and in various technological and biotechnological applications since ancient times [19,20,21,22,23,24]. Of the different biological activities reported for EOs in humans, anti-inflammatory properties appear with a prominent role, presenting potential for a less invasive aid to more orthodox drugs against classical and current illnesses (such as COVID-19) [25,26,27,28,29]. For the above reasons, the development of an inexpensive, environmentally safe and sufficiently accurate method to profile the molecular content and its relative variances of the EOs from *M. urundeuva* seeds and a first approach to obtain insights about such activity are both valuable goals.

Solid-phase microextraction (SPME) is a technique that has been substantially successful in profiling volatile compounds with a low economic and environmental burden [30,31]. SPME consists of the analyte partitioning between the sample and an extracting microcomponent, constituting a polymeric phase that can be solid or liquid that involves a fused silica fiber [32]. Relatively to the advantages of this technique, it is noteworthy that it does not demand sophisticated analytical tools, in addition to organic solvents dispensing and enabling the reuse of the extraction fibers [33,34,35,36]. This technique also presents itself widely dependent on the type of fiber. It was shown in the work of [36] that the PDMS/DVB fiber allowed the identification of more than twice the volatile compounds than the DVB/CAR/PDMS fiber for the pulp of *Eugenia klotzchiana* O.

**Figure 1:**
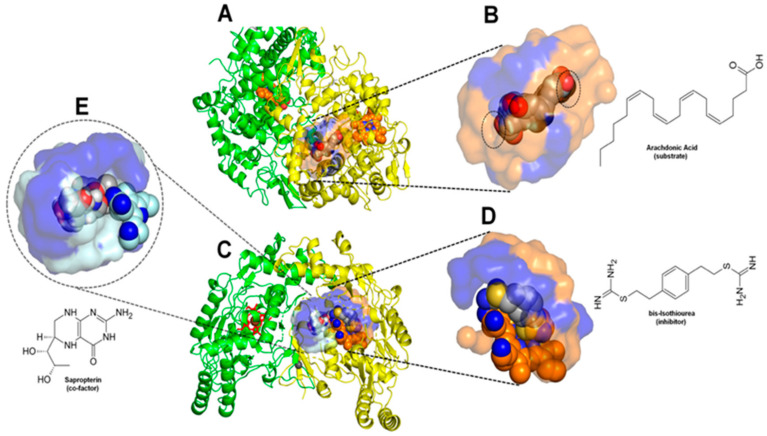
Physical-chemical characteristics for each docking site and their complementarity to the respective higher affinity controls. (**A**)—Full vision of the COX-1 with their two monomers (cartoons in *green* and *yellow*), cofactors (spheres and sticks) and the docked active site in surface and spheres. (**B**)—inlet vision of the same active site containing the crystallographic and the three first docking poses of the more positive control (substrate arachidonic acid represented in spheres, colored in *grey* and *red* for carbons and oxygens, respectively), as well its skeletal formula. The polar residues at the active site (a.s.) surface are shown in *blue*, while the nonpolar in *orange*. Dashed circles show the two a.s. entrances. (**C**)—Similar full vision of the two monomers from iNOS. (**D**)—Inlet of the iNOS active site (iNOSas) containing the more positive control bis-isothiourea (competitive inhibitor). Colors and schemes similar to B, with the heme group from the a.s. also shown in spheres, as well the ligand’s sulfurs and nitrogens, respectively in *yellow* and *blue*. (**E**)—inlet of the iNOS cofactor site (iNOScs) containing the more positive control sapropterin (cofactor). Colors and schemes similar to D, but with the nonpolar regions of the cofactor site (c.s.) in *silver*.

Regarding the research of anti-inflammatory targets for natural compounds, cyclooxygenases (COX) and inducible nitric oxide synthase (iNOS) are two canonical targets [37,38]. The cyclooxygenase enzyme has two isoforms called COX-1 and COX-2, reported in the literature [39,40,41]. Both cyclooxygenases have been already proved as targets of Eos and other natural compounds with anti-inflammatory properties [42,43,44,45]. In fact, the COX natural substrate, arachidonic acid, is a 20-carbon polyunsaturated fatty acid (Figure 1A,B). It is hence expected that the natural adaptation of the enzyme active site to this huge and hydrophobic substrate can also promote a substantial fit to a set of equally bulk and hydrophobic terpene derivatives (overall sesquiterpenes and sesquiterpenoids) present in EOs. Constantly expressed, Cyclooxygenase 1 (COX-1) is constitutive in most tissues and is extremely important to maintain their normal physiological state [46].

The iNOS enzyme produces nitric oxide (NO) in a sustained manner and is expressed in many inflammatory conditions [47,48]. In particular, the participation of this enzyme on the inflammatory and angiogenic mechanisms implicated on tumor growth and carcinogenesis (with both enhancing as inhibiting paradoxical behaviors) has made it a promising and intriguing therapeutic target [49,50,51,52]. To find efficient and safe iNOS inhibitors persists as a difficult task, with just the considerably cytotoxic bis-isothiourea showing significant inhibitory potency currently (Figure 1C,D) [53]. However, a set of natural substances, between them phytochemicals and EO compounds, have shown direct and/or indirect inhibitory effects on this enzyme [54,55].

In addition, iNOS function is possible to modulate both by direct binding at its active site (iNOSa.s.) and by allosteric binding to its coactivator site (iNOSc.s.), i.e., by some substance competing to the binding to this site with the enzyme cofactor tetrahydroneopterin, as shown in Figure 1C,E [56]. This makes this enzyme an interesting and promising double target for EOs compounds.

Computational and chemoinformatics screening approaches have proved to be useful in natural bioactive compounds selection, as well as in the elucidation of which physicochemical characteristics are most related to their structure–activity relationship [57,58]. In particular, such applications have been used successfully for COX-1 and iNOS [58,59,60].

In the present work, we used the SPME technique to identify the volatile compounds (VOCs) present in EOs from aroeira seed samples from different regions of the state of Minas Gerais (Brazil). The bibliographical research for the present work did not find works that had the aroeira seeds as a target for the extraction and evaluation of volatile compounds, whereas works that prioritized the leaves, stems, and bark for this purpose were found. Therefore, we strategically chose the seeds as the object of study due to the lack of information based on the seeds chemical profile. Moreover, we complement them with virtual screening techniques to verify promising compounds to bind to human COX-1 and iNOS, followed by chemical descriptors and a chemoinformatics analysis to understand which characteristics could make them more likely to interact with these canonical anti-inflammatory targets. For iNOS, both affinity with the active site (iNOSa.s.) and with the cofactor site (iNOSc.s.) were verified. The results point to a high incidence of promising compounds for both enzymes. For COX-1 a higher direct correlation between the in silico recovered affinity and the molecular volume and hydrophobicity was found, making the sesquiterpene fraction stand out on the binding. For the iNOS binding at the iNOSa.s., some correlation is still observed between the affinity and molar volume/hydrophobicity, although with considerably less significance compared to COX-1, both sesquiterpenes and monoterpenes, as well a minor fraction of oxygenated compounds, figurating between the higher affinity hits. Finally, for the iNOSc.s. binding, no significant correlation is observed with any simple chemical descriptor, indicating a more complex structure–activity relationship. A relatively weak correlation was found, however, between the affinity and the molecular length and extensibility. Beside this, no sesquiterpenes were found between the higher affinity compounds, the top hits being equally distributed between monoterpenes and oxygenated molecules. This indicates a less permissive site to substantially voluminous and highly hydrophobic molecules. The computational analysis of the more relevant contacts with each respective active site shed light on the above mentioned chemical descriptor features, in addition to the support found on the comparison with experimental structures of the targets bound to canonical ligands.

Taken together, the results of our study illustrate the applicability of SPME profiling added to chemoinformatics analysis to obtain fast, environmentally friendly and non-expansive information about the medicinal and biotechnological potential of natural sources. It is hoped that the results presented here will be useful in directing future prospecting approaches for natural bioactive compounds, as well as rational planning for new ligands.

## 2. Results

### 2.1. Solid Phase Microextraction Allows Fine Profiling of the Aroeira Seeds Volatiles

**Figure 2:**
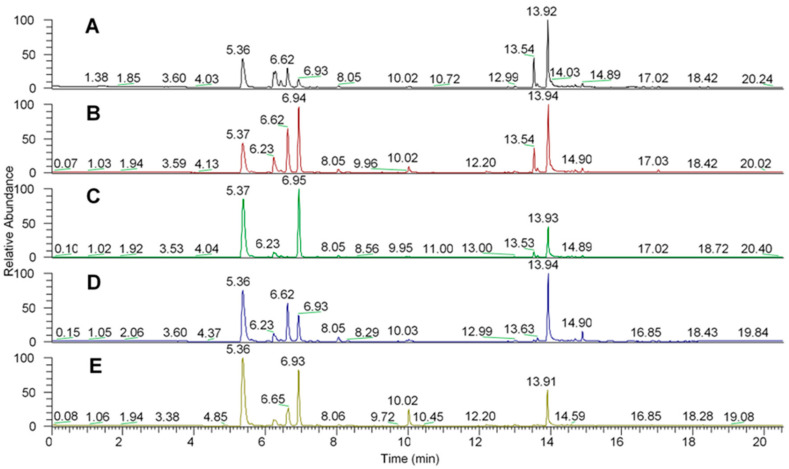
Chromatograms showing DVB/PDMS fiber VOC extraction results. Each chromatogram listed from (**A**–**E**) represents, respectively, matrices 1 to 5.

Figure 2 shows the chromatograms of the seed samples of all aroeira trees analyzed.

The peaks show the volatile organic compounds identified in a *m*/*z* ratio.

As it can be seen, all chromatograms show similarities between the peaks in relation to retention time, because even though they are chromatograms of different trees, they are still the same species which do not differentiate significantly in the synthesis of secondary metabolites, since these are determined primarily by the genotype.

Table 1 shows the HS-SPME/GC-MS identified compounds through the chromatograms of the samples.

Among the compounds found in the aroeira samples using DVB-PDMS fibers, 22 volatile organic compounds of different chemical classes were identified, including terpenoids, carboxylic acids, and ketones. With 17 compounds identified, terpenoides were the most found in all samples, especially caryophyllene and 3-carene. When analyzing the literature of similar works in the characterization of aroeira’s chemical profile, chemical compounds such as 1 R-α-pinene, 3-carene, caryophyllene, camphene, and limonene were also frequently found, such as in the work of [12]. In addition to being identified in all five samples, 3-carene was the major constituent in samples 3, 4 and 5, with an abundance of 46.5%, 49.9%, and 55.2%, respectively, and the second most abundant in sample 2 (34.8%). [38] also identified 3-carene as the most common monoterpene (30.4%) when the activity of the essential oil of the “red aroeira” (*Schinus terebinthifolius* Radd) was evaluated as an antibacterial agent.

In the work of [61], two samples of aroeira regarding the profile of their essential oils were analyzed. One sample was from Mato Grosso, and the other from Tocantins, and both presented 3-carene as a major constituent (78.1%; 56.3%), which is in line with the present work, where all the samples comprised this compound, which was also a major contributor in the composition of most samples (samples 3, 4, and 5). However, in the work by [30] where the chemical profile of aroeira and “red aroeira” (*Schinus terebinthifolius* Radd) was evaluated, the constituent 3-Carene was not found, and trans-geranylacetone was the major compound found. Therefore, it is evident that although samples from different regions show differences in the composition of volatile compounds, there is still a predominance of certain terpenes that characterize the species. Regarding monoterpenes found in this work, it is worth mentioning that the essential oils of the aroeira tree are pointed out in other papers as antimicrobial agents due to the action of some compounds, such as 3-carene, a monoterpene involved in the bactericide action on wild-type hospital strains [62]. Ref [63] found the monoterpenes α-pinene and D-limonene as compounds with a higher antibacterial activity against gram-negative and gram-positive bacteria in the constitution of the essential oil of two species of the genus Schinus (Anacardiaceae).

### 2.2. Comparative Virtual Docking Points for Significant Distinctions between Hits for COX-1 and iNOScs, with iNOSas Presenting an “Intermediary” Behavior between Both Sites

**Figure 3:**
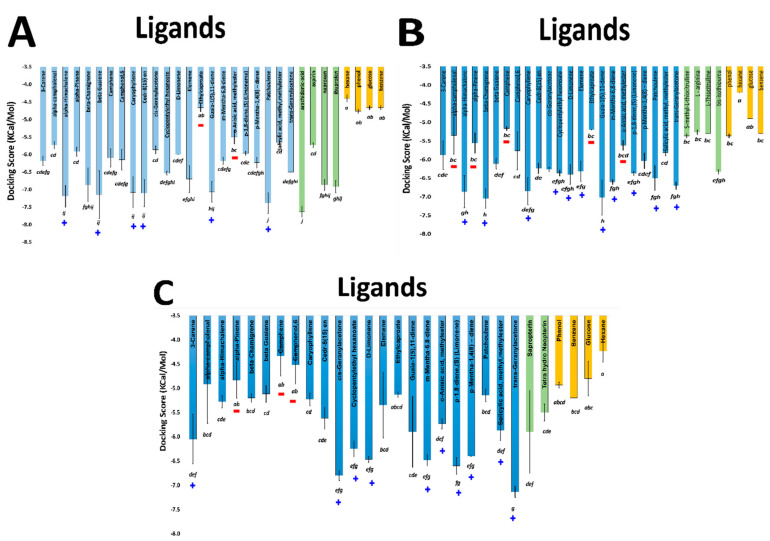
Average docking scores for the three first poses of the respective aroeira compounds and controls at the three targeted sites. (**A**)—COX-1; (**B**)—iNOS active site (iNOSas); (**C**)—iNOS cofactor site (iNOScs). Bars are colored in blue for the aroeira compounds, green for positive controls and yellow for negative controls. Letters below the bars indicate the grouping of the scores according to ANOVA. A blue “plus” (+) and a red “minus” (−) symbols indicate, respectively, top and worst hits between the aroeira volatiles for each site at each enzyme.

**Figure 4:**
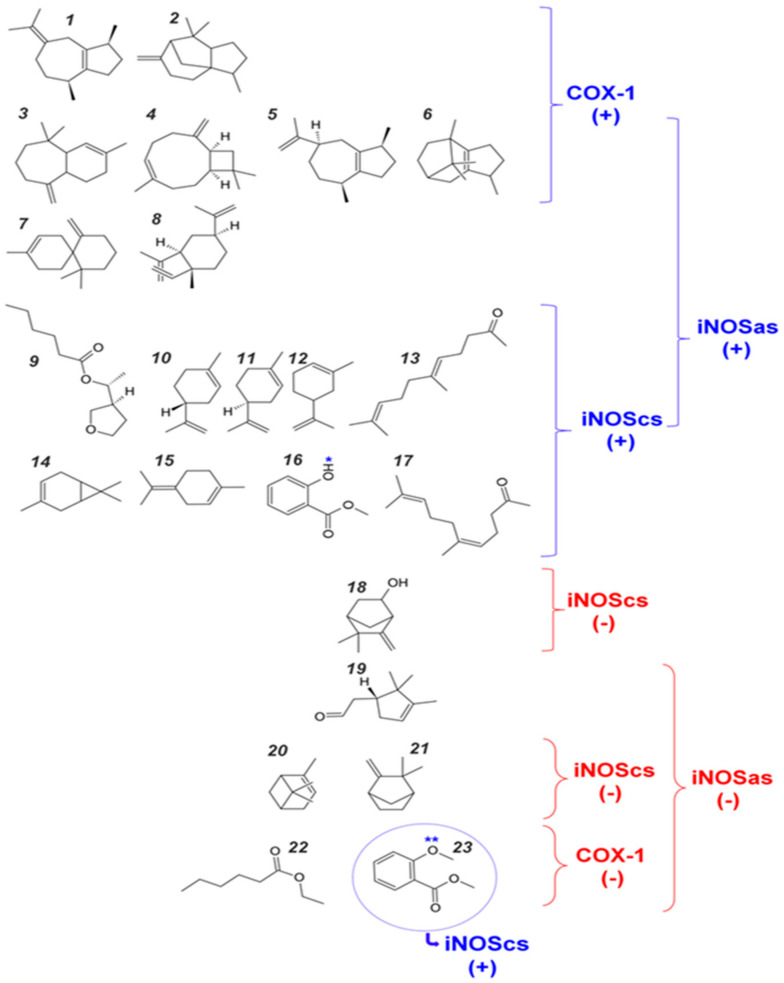
Aroeira compounds and respective classifications according affinities to eachsite. **1**—β-guaiene; **2**—Cedr(8)-15 ene; **3**—α-Himachalene; **4**—Caryophyllene; **5**—Guaia-1(5),11-diene; **6**—Patchoulene; **7**—β-Chamigrene; **8**—Elemene; **9**—Cyclopentylethylhexanoate; **10**—D-Limonene; **11**—p-1,8-diene,(S)(Limonene); **12**—m-Mentha-6,8-diene; **13**—trans-Geranylacetone; **14**—3-Carene; **15**—p-Mentha-1,4(8)–diene; **16**—Salicylic acid, methyl, methylester; **17**—cis-Geranylacetone; **18**—Camphenol,6; **19**—α-Campholenal; **20**—α-Pinene; **21**—Camphene; **22**—Ethylcaproate; **23**—o-Anisic acid, methylester. The two salicylate derivatives (compounds **16** and **23**), potentially able to suicide inhibition (directly, or after modifications) are highlited with a blue asterisk.

These compounds were compared against the compounds recovered by the experimental assays (Figure 3—blue bars). As expected, the negative controls presented an intermediary or low binding affinity in all target sites. The positive and negative controls are clearly distinguishable for COX-1and iNOScs, but iNOSas presented a mixing behavior, except bis-isothiourea (a known competitive inhibitor) and hexane which presented respectively a high and low affinity to the active site compared to the other controls. Thus, we sorted the tested compounds into three groups based on how close their docking scores were from the best positive control score (top compounds group), from the worst negative control score (worst compounds group) or compounds with intermediary behavior (middle compounds group). Once the groups were defined independently for each target site, we looked closer to the molecular structure of these compounds searching for possible structural patterns (Figure 4). Perhaps predictably, no superposition of compound structures was found between COX-1 and iNOScs once they were at quite distinct sites. Otherwise, iNOSas selected multiple top compounds able to bind favorably both in COX-1 and iNOScs, suggesting possible multi-front anti-inflammatory compounds. Between these, stand out compounds as caryophyllene and patchoulene (respective compounds **4** and **6** in Figure 4), already with substantially reported anti-inflammatory activities [25,26,27,28,29]. Similarly, a set of monoterpenes and oxygenated compounds were recovered as top hits for iNOS by binding to both sites, the active and the cofactor one. In this way, the iNOSas pocket has shown an intermediary behavior concerning compound selectivity compared to the other two sites here studied. The same was observed for the worst groups, highlighting the compound **23** which was the only one to present a low affinity to COX-1 and iNOSas and a high affinity to iNOScs, suggesting a potential specific inhibitor.

### 2.3. Volume and Hydrophobicity as Major Components for the COX-1 Affinity, Followed by Inos and with iNOScs Presenting a More Complex and Less Predictable Behavior

**Figure 5:**
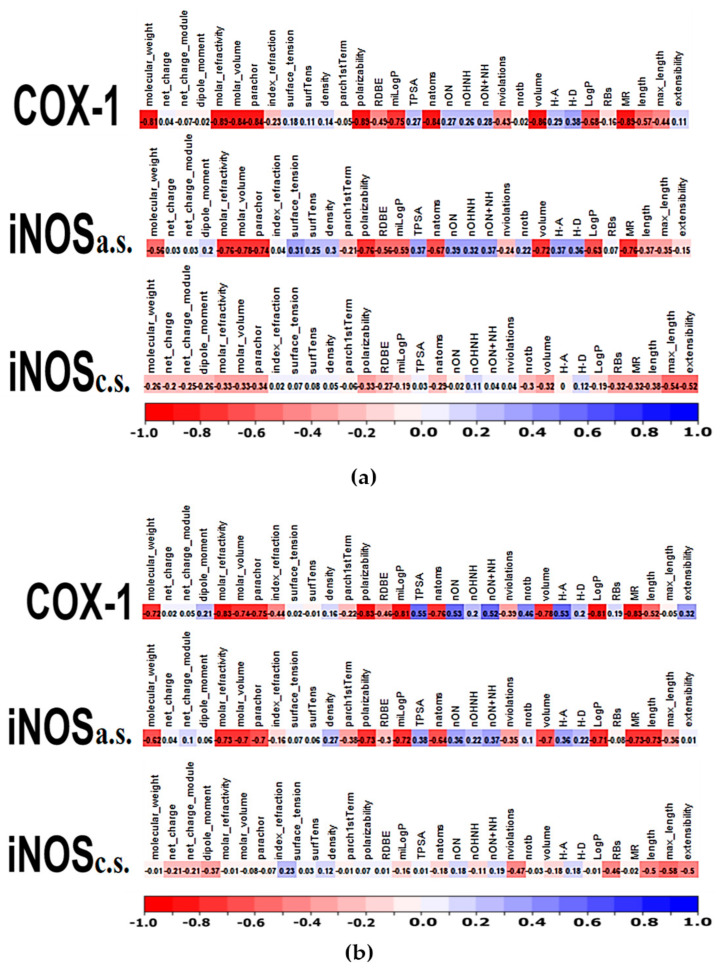
(**a**) **Aroeira + Controls**: Correlation vectors between different molecular descriptors from the aroeira compounds and their docking scores at each site. Negative correlations between the descriptors and the dockins scores (red) indicate attributes that improve the affinity. Positive correlations (blue) indicate attributes that draw back the affinity. No correlation (white) indicates no significant influence. (**b**) **Aroeira**: Correlation vectors between different molecular descriptors from the aroeira compounds and their docking scores at each site. Negative correlations between the descriptors and the dockins scores (red) indicate attributes that improve the affinity. Positive correlations (blue) indicate attributes that draw back the affinity. No correlation (white) indicates no significant influence.

**Figure 6:**
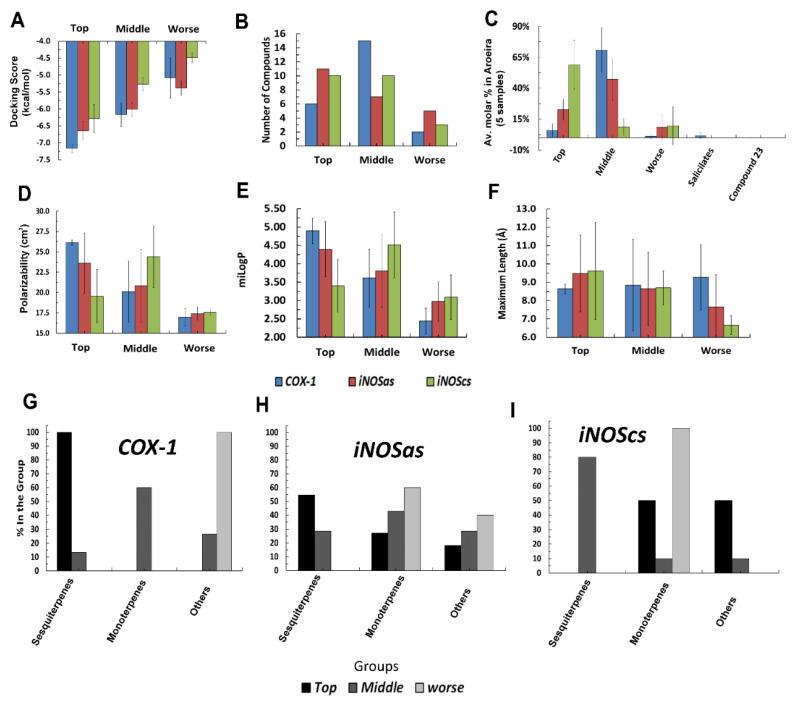
Statistics and numbers concerning the aroeira compounds classified according to their respective virtual affinities for the three targeted sites. (**A**–**F**)-respective plots of the docking scores, number of compounds (between the 23 profiled), average percent of the compounds at the five samples, average polarizability, miLogP and maximal length, according to the compounds belong to the *top*, *middle*, or *worst* groups for COX-1, iNOSas and iNOScs. (**G**–**I**)-Respective plots for COX-1, iNOSas and iNOScs of the percent of sesquiterpenes, monoterpenes and other classes at each one of the *top*, *middle*, and *worst* docking score classifications.

The in silico recovered affinity for aroeira compounds to the COX-1 active site suggests to be directly dependent on volume and hydrophobicity. This can be inferred both by the fact that its top hits are totally composed of voluminous sesquiterpenes, as by the substantial anti-correlation between a set of chemical descriptors related to volume and hydrophobicity (as molar volume, polarizability, molar refractivity, LogP, parachor) and their docking free energy (Figure 4, Figure 5 and Figure 6). Also, the considerable positive correlation between the docking free energy and descriptors related to polarity and hydrogen bonds, as total polar surface area (TPSA) and the number of hydrogen bond acceptors, corroborates this trend (Figure 5).

### 2.4. Structural Interpretation of the Differential Selectivity for COX-1 and iNOS Active/Cofactor Sites for the Different Ligand Classes on Aroeira Seeds

In order to better understand the correlations between the target selectivity and the aroeira VOCs physical-chemical attributes, we carried structural analysis on the contact patterns between the top hits at each active site. We also compared the contact patterns from the docked VOCs and from the respective first ranked positive controls (from experimental and docked structures) in order to best validate our results.

**Figure 7:**
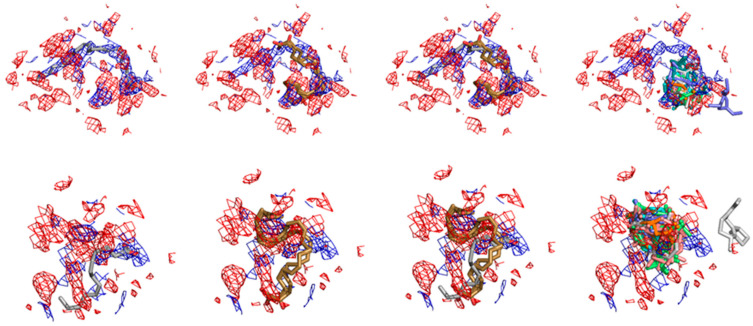
Different binding modes for the ligands at the COX-1 crystallographic and docked structures and their fits to crystal electronic density. At the *Top* the electronic densities and different molecular model superpositions for the human COX-1 complexed with arachidonic acid structure at the PDB-ID:1DIY is shown. At the *bottom*, the superpositions with the electronic density of the same complex as solved at the PDB-ID:1U67 are depicted. In both cases, the *red* and *blue meshes* depict the respective F_o_F_c_ electronic density (i.e., electronic densities for which the authors model have found atomic fitting) and 2F_o_F_c_ maps (i.e., electronic densities for which the author’s molecular model has not found atomic superposition). In both cases also the respective electronic density maps are considered with a σ factor of 1.0. From *left* to *right* it can be seen the respective superposition at both densities for the models deposited at the *protein data bank*; for our Arachidonic acid docked structures (positive control); for both (crystallographic and docked); for the set of the three first docked poses of the top hits for this enzyme between the VOCs profiled from the *M. urundeuva* seeds.

A primary point to be discussed here is that the first ranked docking positive control for COX-1 (the substrate arachidonic acid) does not reproduce the poses of the crystallographic models at the two available PDB structures, the PDB-ID:1DIY (with 3.00 Å resolution) and the PDB-ID:1U67 (with 3.10 Å resolution) (Figure 7). This is not surprising, however, when both the resolution and the specific electronic density map inside the enzyme active site of each crystal structure are taken into accounts.

**Figure 8:**
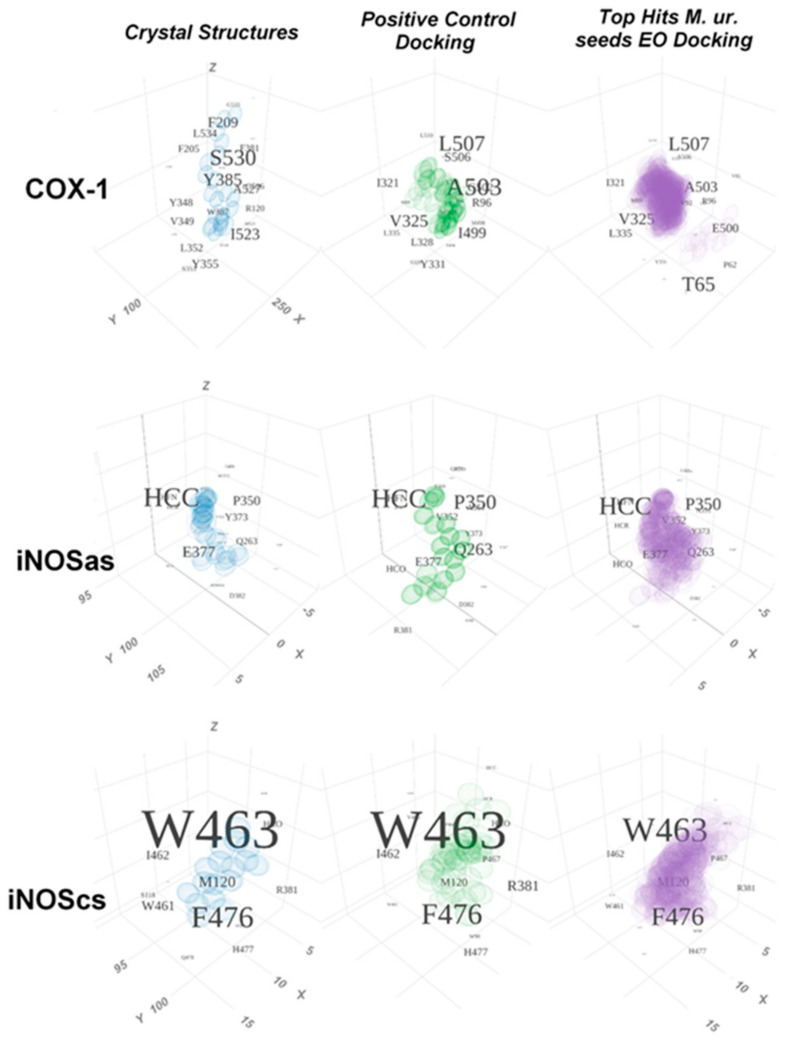
Tridimensional graphic representation of the major contacts at the active site for positive controls and top compounds from essential oils from M. urundeuva seeds. The tridimensional representation (at the x, y, z axes) of each respective active site is arbitrarily centralized at the geometrical center of the set of atoms involved in contacts around all the analysis. The grid spacement at the three-dimensional space in each graphic is always of 5 Å at each dimnension. The density of the spheres on the image depicts how many superposed atoms there are at that region between different docks with the same ligand (the three first poses from the docking procedure were considered) and between different compounds. The font size depicting the residue identity and number increases according to the average intensity of the contacts involving it (i.e., the average contact area superposition between the ligands and the residue). The subdivision of the large Heme group was carried out as mentioned in the Materials and Methods section. From top to bottom it can be noticed the contacts for the COX-1, iNOSa.s. and iNOSc.s. pockets. From the left to the right the respective contact intensities for the crystallographic controls (the arachidonic acid contacts at the PDB-ID:1DIY for COX-1, the L-Arginine-iNOS contacts at the PDB-ID:3NOS plus the isothiourea-iNOS contacts at the PDB-ID:4NOS for iNOSa.s., the tetrahydroneopterin-iNOS contacts at the PDB-ID:4NOS for iNOSc.s.); for the docked three first poses for the most positive controls (arachidonic acid for COX-1, bis-isothiourea for iNOSa.s. and tetrahydropterin for iNOSc.s.); as well the three first poses of the top hits for each site are depicted. *M. ur.* = *Myracrondruon urundeuva*. *EO* = Essential oil.

In Figure 8, three-dimensional maps considering the intensity (the sphere size) and frequency between different ligands and poses (the font size) for the major contacts at each target are comparatively depicted. The contacts are compared (from left to right) between crystallographic poses (arachidonic acid at the PDB-ID:1DIY for COX-1; L-arginine at PDB-ID:3NOS and isothiourea at PDB-ID:4NOS for iNOSas; sapropterin at PDB-ID:4NOS for iNOScs), the docked three first poses of the respective most positive controls (arachidonic acid for COX-1; bis-isothiourea for iNOSas; sapropterin for iNOScs) and the set of three first poses for all the top compounds for each target.

### 2.5. Two Salicylate Derivatives on Aroeira Seeds Seems to Be Promissor for Direct, or after Modification, Suicide Inhibition of the COX-1 Enzyme

**Figure 9:**
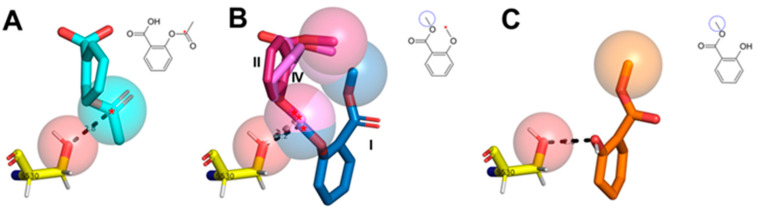
Docking poses for acetylsalicylic acid and salicylate related compounds from aroeira seeds at COX-1 active site. (**A**)-Acetylsalycilic acid (aspirin^TM^, positive control); (**B**)-Aroeira compound **23** (the respective docking poses I, II and IV are depicted); (**C**)-Aroeira compound 16. The contacts between the S530 residue (subjected to suicide inhibition by aspirin^TM^) and the compound’s nucleophilic attack sensitive center (when present) or hydroxyl radical are both shown as dashed lines. Both at the skeletal formulas as at the docked structures, the respective groups susceptible to S530 nucleophilic attack (*red* asterisk/sphere) and esterified to the original acid (dashed circle/sphere) are, all of them, highlighted when present.

Two salicylate derivatives (compounds **16** and **23** in Figure 4) are present in minor concentrations at the VOCs from M. urundeuva seeds (Figure 6). Although they are present in substantially low concentrations and even absent in some samples, the docking poses recovered by these two compounds trend to approximate the respective methoxy and hydroxyl groups from the COX-1 catalytic serine on similar way that the suicide acetyl in aspirin^TM^ (Figure 9).

## 3. Material and Methods

Mature seeds of *Myracrodruon urundeuva* from 5 adult matrices were collected in October and November 2019 in different areas of Sete Lagoas-MG, Brazil, located at coordinates 19°28′29.0″ S 44°11′39.9″ W, at an altitude of 751 m. According to Köeppen, the regional climate is Cwa, i.e., typical savanna climate, with dry winters and wet and rainy summers [61]. The seeds of each matrix were transferred separately to the chemistry laboratory, where the manual process of removing dirt particles and undesirable parts of the plant was carried out. The next step consisted of grinding the seeds of each matrix separately in an A 11 IKA analytical mill, followed by weighing each sample on a Marbeg balance and storing it in headspace flasks.

Headspace solid phase microextraction (HS-SPME) was employed for the extraction of volatile compounds, using a semi-polar polymeric film, polydimethylsiloxane-divinylbenzene (PDMS/DVB). In the extraction of the VOCs, 2 g of the previously ground seeds were used, placed in a 20 mL headspace vial, the containers were closed with an aluminum seal and a rubber septum. The 20-mL headspace vial was then placed on an aluminum block and heated to 60 °C. After 5 min of heating, the SPME polymeric film (PDMS/DVB) was exposed to the sample for 20 min, and then the holder containing the polymeric film was retracted and manually inserted into the injector of the gas chromatograph coupled to the mass spectrometer, exposing the polymeric film during 5 min for the desorption of the extracted volatile organic compounds. The two figures below show aroeira seeds in early stages of development.

### 3.1. Gas Chromatography—Mass Spectrometry

Aroeira seed samples were analyzed by a gas chromatograph (Trace GC Ultra) coupled to a mass spectrometer (Polaris Q model, Thermo Scientific, San Jose, CA, USA), with an ion trap type analyzer, located in the Mass Spectrometry Laboratory of the UFMG’s Chemistry Department. The samples were analyzed in the following settings: injector temperature of 250 °C; splitless mode injection, desorption time of 5 min; injector temperature of 200 °C; interface temperature of 275 °C. The column heating temperature was set up starting at 40 °C and remaining at the temperature for 1 min followed by gradual temperature increase of 10 °C/min up to 100 °C keeping the isotherm for 1 min, 12 °C/min up to 150 °C, keeping the isotherm for 1 min and then 15 °C/min up to 245 °C, temperature at which the isotherm was kept for 1 min. The detector was kept in scanning mode (fullscan, from 35 to 300 *m*/*z*), using the Electron Impact Ionization (EI) technique, at an energy of 70 electron-volt (eV). Throughout the process an HP-5 MS capillary chromatographic column (5% phenyl and 95% methylpolisiloxane) was used, of the following dimensions: 30 m in length, 0.25 mm (mm) internal diameter and 0.25 µm film thickness [33,35].

### 3.2. Volatile Compound Identification

For the identification of the volatile compounds, the mass-to-charge ratio (*m*/*z*) corresponding to each peak generated by the chromatogram was compared with the mass spectra obtained through ionization by EI, using energy of 70 eV, and the fullscan range from 35 to 300 *m*/*z* As such, the mass spectra of the analytes found were compared with the mass spectra data obtained from the NIST (National Institute of Standards and Technology) library, using as an auxiliary tool the data recorded in the literature for the confirmation of the volatile compounds found in the seed samples. The RSI index consists of a numerical comparison factor, where the higher the value, the closer the compound is to the one found in the NIST library. However, only peaks with values of relative standard intensity (RSI) higher than 600, and a signal-to-noise ratio (S/N) above 50 decibels were selected. Intensity values of the peaks obtained and the S/N ratio were obtained using Thermo Electron Corporation’s XCalibur 1.4 program and the data were transferred to Microsoft Office Excel 2013, where the peak selections were made according to the S/N ratio in the UFSJ/CSL Chemistry Laboratory.

### 3.3. Virtual Docking Assays

Structure- based virtual screening applying docking simulations was performed using the AutoDock Vina tool [64]. The respective structures for each target were obtained from the *protein data bank* [65] or modeled by homology. The human COX-1 was modeled from the correspondent human sequence obtained from UniProt [66] using the tool Swiss-Model [67] and the ovine structure at the PDB-ID:1DIY (originally complexed to the arachidonic acid substrate). The human iNOS structure was obtained from the PDB-ID:4NOS (originally complexed the inhibitor isothiourea and containing the tetrahydroneopterin cofactor) using the Swiss-Model tool to fill small gaps at the protein crystal construction. For this enzyme, two docking procedures were carried out for each ligand: one at the enzyme active site (here called iNOSa.s.) and the other at the cofactor site (here called iNOSc.s.). In all the three cases, the enzyme dimmeric structure was used and the crystallographic ligand was removed before the docking procedures, conserving just the protein dimmer and the respective co-factors (heme groups for both enzymes, in addition to zinc ion and both tetrahydroneopterin for iNOS at the iNOSa.s. docking and just heme, zinc ion and the tetrahydroneopterin at the non-docked monomer for this enzyme at the iNOSc.s. docking procedure).

The docking boxes for both targets were centered at the position originally occupied by the respective crystallographic ligands (the arachidonic acid from the PDB-ID:1DIY for COX-1; the isothiourea ligand for the iNOSa.s. docking in iNOS and the tetrahydroneopterin cofactor for the iNOSc.s. docking at this enzyme). The box dimensions were planned in order to preserve enough of the crystallographic context about 8 Å around different crystall ligands previously inspected by the present authors for each enzyme, finishing on a common x, y, z set of dimensions of 17, 25, 17 Å, respectively.

A ligand virtual dataset was composed of volatile compounds of aroeira determined by the mass spectrometry method above. MOL2 files from the ligands were downloaded from PubChem [68]. As negative controls for the virtual docking procedures, we chose four non-druggable molecules concerning these enzymes (glucose, hexane, benzene, and phenol) for all the three pockets. Specific positive controls were chosen for each pocket: the substrate arachidonic acid, the suicide inhibitor acetyl-salicylic acid and the competitive inhibitors naproxen and ibuprofen for COX-1; the substrate *L*-Arginine and the competitive inhibitors *S*-methyl-*L*-thiocitruline, *L*-thiocitruline and bis-isothiourea for the iNOSa.s. pocket in iNOS; the cofactor tetrahydroneopterin and its derivative sapropterin for the iNOSc.s. pocket in this same enzyme. This virtual ligand dataset was prepared using MGLTools [69] and automatically docked through AutoDockVina tool usign Python housemade scripts. The docking procedures were carried with an exhaustiveness parameter of 128, in order maximize the sampling and accuracy. For each system, the first three docked poses were taken to score and structural analysis. The docking results were analyzed using housemade scripts and the tool PyMOL [70].

The three first poses considering the docking affinity scores were taken from each ligand and ANOVA was used for statistical analysis and mean comparison as determined by Tukey’s HSD (honestly significant difference) test. The R language and computational environment was used for all the statistical analyses [71].

### 3.4. Molecular Descriptors and Statistical Analysis

Chemoinformatics molecular descriptors for the volatile compounds of aroeira were obtained from the free tools: ACD/ChemSketch [72], Molinspiration platform [73] and 3D-QSAR.com platform [74]. Correlogram among different molecular descriptors and docking score affinities were calculated using R. The Pearson correlation coefficient was determined.

### 3.5. Estimation of the Major Contacts Involved in Ligand-Target Interactions

The contact areas were computed according to the methodology described in [75]. Basically, this area is computed for each pair of atoms at the ligand-target complexes. It is equivalent to the excluded or untouched area of a rolling water molecule (probe). Thus, a null contact area indicates that one (or more) water molecule could be interposed between two atoms, defining a potential cavity in terms of this heuristics. It also indicates how tightly packed a group of atoms is in space, given that the larger the contact area, the better spatially clustered they will be. As a consequence, atoms with non-zero contact areas delimit an interface region between any sets of different biomolecules in close contact.

We represented three-dimensionally the interaction intensity at each residue/subsite at the pocket with dot spheres designating superimposed ligand atoms as a result of the successive docking into the respective targets. Letters and numbers represent target residues positioned according to the geometric center of its atoms in the ligand-target complex. The font size indicates the average contact area of the residues with such ligands.

As a larger prosthetic group, the HEM group at the iNOS a.s. and c.s. respective pockets (in which the ligand interacts or is able to interact with this group) was divided into parts, with the following names: HFN = the Fe^2+^ and the N from the porphyrins at the center; HCC = SP2 carbons of the porphyrin ring; HCR = SP3 carbons branched from the porphyrin ring that support the carboxylic groups; HCO = oxygens of carboxylic groups at the branch ends. The HEM groups were considered as part of the targets.

## 4. Conclusions

It was possible to observe that HS-SPME, a method considered green for not using organic solvents and requiring minimum sample preparation, was efficient in the extraction of volatile compounds from aroeira seed samples collected in five trees of different areas in Sete Lagoas- MG, where it was possible to find and identify 22 volatile organic compounds of different chemical classes, among terpenoids, carboxylic acids, and ketones. 3-carene was present in all samples as the main constituent in three of the five samples.

The chromatography technique used was efficient in the separation process of volatile organic compounds, coupled with the analysis in mass spectrometry which allowed the identification of these compounds.

## Figures and Tables

**Table 1:** Volatile compounds extracted from Aroeira (*Myracrodruon urundeuva*) seeds by HS-SPME/GC-MS.

N°	Volatile Organic Compoud	CAS	Formula	Sampled Trees				
				A.1	A.2	A.3	A.4	A.5
**MONOTERPENES**								
1	*α*-Pinene ^a,b,c,d,e,f,g,i,j,l,m,n^	7785-70-8	C_10_H_16_	22.6%	ND	ND	ND	ND
2	3-Carene ^a,b,c,d,f,g,i,k,l^	13466-78-9	C_10_H_16_	11.0%	34.8%	46.5%	49.9%	55.2%
3	Camphene ^f,i,l,m,n^	79-92-5	C_10_H_16_	ND	ND	0.5%	0.6%	0.5%
4	*α*-Campholenal ^f^	4501-58-0	C_10_H_16_O	ND	7.7%	ND	4.6%	ND
5	Camphenol,6-	3570-04-5	C_10_H_16_O	14.9%	ND	4.2%	ND	5.7%
6	D-Limonene ^a,b,c,d,e,f,g,h,l,n^	5989-27-5	C_10_H_16_	ND	23.0%	ND	ND	ND
7	* m * -Mentha-6,8-diene^®^	1461-27-4	C_10_H_16_	ND	ND	30.7%	ND	ND
8	p-Mentha-1,4(8)—diene ^d,f,k^	586-62-9	C_10_H_16_	1.0%	1.3%	0.9%	2.9%	ND
9	p-1,8-diene,(S)	5989-54-8	C_10_H_16_	5%	ND	ND	10.4%	21.1%
	**SESQUITERPENES**							
10	* β * -Guaiene ^k^	88-84-6	C_15_H_24_		0.8%			0.2%
11	Caryophyllene ^b,c,d,e,f,g,i,j,m,n^	87-44-5	C_15_H_24_	11.4%	6.0%	1.9%	0.6%	0.3%
12	Cedr-8(15) ene	11028-42-5	C_15_H_24_	1.7%	1.2%	0.6%	2.6%	ND
13	Elemene ^d,f,m,n^	339154-9	C_15_H_24_	0.2%	ND	ND	ND	ND
14	*β*-Chamigrene	18431-82-8	C_15_H_24_	ND	ND	ND	ND	0.4%
15	Guaia-1(5),11-diene	3691-12-1	C_15_H_24_	ND	ND	0.4%	ND	ND
16	Patchoulene	1405-16-9	C_15_H_24_	ND	0.6%	ND	ND	ND
17	1H-Benzocycloheptene,2,4aα,5,6,7,8,9aα-octahydro-3,5,5-trimethyl-9-methylene-	3853-83-6	C_15_H_24_	1%	0.8%	ND	0.8%	ND
**OTHER CLASSES**								
18	Ethylcaproate	123-66-0	C_8_H_16_O_2_	4.0%	ND	0.8%	ND	ND
19	* Cis- * Geranylacetone	3879-26-3	C_13_H_22_O	27.2%	ND	25.4%	24.3%	10.3%
20	o-Anisic acid, methylester	606-45-1	C_9_H_10_O_3_	ND	0.5%	ND	ND	0.7%
21	Salicylic acid, methyl, methylester ^e^	119-36-8	C_8_H_8_O_3_	ND	2.3%	ND	2.0%	5.6%
22	*Trans*-Geranylacetone	3796-70-1	C_13_H_22_O	ND	21.6%	ND	ND	ND
23	Hexanoic acid 1-cyclopentylethylester	NA	C_13_H_24_O_2_	ND	ND	ND	0.6%	ND

Identified Volatile compounds: letters indicate compounds found by other authors in different species of the Anarcadiceae family.

## Data Availability

All data is contained within the article.

## References

[1] Nunes Y.R.F., Fagundes M., Almeida H.S., Veloso M.D.M. (2008). Aspectos ecológicos da aroeira (*Myracrodruon urundeuva* Allemão-Anacardiaceae): Fenologia e germinação de sementes. Rev. Árvore Viçosa.

[2] Feliciano A.L.P., Maragon L.C., Holanda A.C. (2008). Morfologia de sementes, de plântulas e de plantas jovens de aroeira (*Myracrodruon urundeuva* Allemão). Rev. Biol. Ciências Terra Paraíba.

[3] Virgens I.O., Castro R.D., Fernandez L.G., Pelacani C.R. (2012). Comportamento fsiológico de sementes de *Myracrodruon urundeuva* fr. all. (Anacardiaceae) submetidas a fatores abióticos. Ciência Florest. St. Maria RS.

[4] Araújo R.D.I. (2017). Atividade Antimicrobiana e Citotóxica de Óleo Essencial e Extratos Orgânicos Provenientes da Myracrodruon urundeuva Fr. Allem. (Aroeira-do-Sertão). Dissertação de Mestrado.

[5] Aquino N.C., Araújo M.R., Silveira R.E. (2017). Intraspecific Variation of the Volatile Chemical Composition of *Myracrodruon urundeuva* Fr. Allem. (“Aroeira-do-Sertão”): Characterization of Six Chemotypes. J. Braz. Chem. Soc..

[6] Medeiros A.C.S., Smith R., Probert R.J., Sader R. (2000). Comportamento fisiológico de sementes de aroeira (*Myracrodruon urundeuva* Fr. All.), em condições de armazenamento. Bol. Pesq. Fl. Colombo.

[7] Silva P.D.D. (2018). Caracterização de Compostos Fenólicos por Espectrometria de Massas e Potencial Antioxidante das Cascas de Myracrodruon urundeuva (Aroeira-do-Sertão) do Cariri Paraibano. Dissertação de Mestrado.

[8] Ferreira P.M.P., Farias D.F., Viana M.P., Souza P.M., Vasconcelos I.M., Soares B.M., Pessoa C., Costa-Lotufo L.V., Moraes M.O., Carvalho A.F.U. (2011). Study of the antiproliferative potential of seed extracts from Northeastern Brazilian plants. Biomed. Sci. An. Acad. Bras. Cienc..

[9] Soares A.M.S., Oliveira J.T.A., Rocha C.Q., Ferreira A.T.S., Perales J., Zanatta A.C., Vilegas W., Silva C.R., Costa-Junior L.M. (2018). *Myracrodruon urundeuva* seed exudates proteome and anthelmintic activity against Haemonchus contortus. PLoS ONE.

[10] Machado A.C., Oliveira R.C. (2014). Phytotherapy medicines in dentistry: Evidence and perspectives on the use of “Aroeira-do-sertão” (*Myracrodruon Urundeuva* Allemão). Rev. Bras. Plantas Med..

[11] Lica I.C.L., Soares A., de Mesquita L.S.S., Malik S. (2018). Biological properties and pharmacological potential of plant exudates. Food Res. Int..

[12] Carvalho M.G., Melo A.G.N., Aragão C.F.S., Raffin F.N., Moura T.F.A.L. (2013). *Schinus terebinthifolius* Raddi: Chemical composition, biological properties and toxicity. Rev. Bras. Pl. Med. Botucatu.

[13] Ceruks M., Romoff P., Favero A.O., Lago J.H.G. (2007). Constituintes fenólicos polares de *Schinus terebinthifolius* Raddi (Anacardiacea). Rev. Química Nova São Paulo.

[14] Jain M.K., Yu B.Z., Rogers J.M., Smith A.E., Boger E.T., Ostrander R.L., Rheingold A.L. (1995). Specifi c competiti ve inhibitor of secreted phospholipase A2 from berries of Schinus terebinthifolius. Phytochemistry.

[15] Santos B.O., Augusti R., Melo J.O.F., Takahashi J.A., Araújo R.L.B. (2020). Optimization of extraction conditions of volatile compounds from pequi peel (*Caryocar brasiliense* Camb.) using HS-SPME. Res. Soc. Dev..

[16] Bergman M.E., Davis B., Phillips M.A. (2019). Medically Useful Plant Terpenoids: Biosynthesis, Occurrence, and Mechanism of Action. Molecules.

[17] Aponso M., Patti A., Bennett L.E. (2020). Dose-related effects of inhaled essential oils on behavioural measures of anxiety and depression and biomarkers of oxidative stress. J. Ethnopharmacol..

[18] Aziz Z.A.A., Ahmad A., Setapar S.H.M., Karakucuk A., Azim M.M., Lokhat D., Rafatullah M., Ganash M., Kamal M.A., Ashraf G. (2018). Essential Oils: Extraction Techniques, Pharmaceutical and Therapeutic potential Review. Curr. Drug Metab..

[19] Da Silva L.L., De Almeida R., Verícimo M.A., De Macedo H.W., Castro H.C. (2019). Atividades terapêuticas do óleo essencial de melaleuca (*Melaleuca alternifolia*) Uma revisão de literatura. Braz. J. Health Rev..

[20] Dhakad A.K., Pandey V.V., Beg S., Rawat J.M., Singh A. (2017). Biological, medicinal and toxicological significance ofEucalyptusleaf essential oil: A review. J. Sci. Food Agric..

[21] Farrar A.J., Farrar F.C. (2020). Clinical Aromatherapy. Nurs. Clin. N. Am..

[22] Hosseinzadeh S., Jafarikukhdan A., Hosseini A., Armand R. (2015). The Application of Medicinal Plants in Traditional and Modern Medicine: A Review of Thymus vulgaris. Int. J. Clin. Med..

[23] Yuan R., Zhang D., Yang J., Wu Z., Luo C., Han L., Yang F., Lin J., Yang M. (2020). Review of aromatherapy essential oils and their mechanism of action against migraines. J. Ethnopharmacol..

[24] Ramsey J.T., Shropshire B.C., Nagy T.R., Chambers K.D., Li Y., Korach K.S. (2020). Essential Oils and Health. Yale J. Biol. Med..

[25] Asif M., Saleem M., Saadullah M., Yaseen H.S., Al Zarzour R. (2020). COVID-19 and therapy with essential oils having antiviral, anti-inflammatory, and immunomodulatory properties. Inflammopharmacology.

[26] Zuo X., Gu Y., Wang C., Zhang J., Zhang J., Wang G., Wang F. (2020). A Systematic Review of the Anti-Inflammatory and Immunomodulatory Properties of 16 Essential Oils of Herbs. Evid. Based Complement. Altern. Med..

[27] Jurenka J.S. (2009). Anti-inflammatory properties of curcumin, a major constituent of Curcuma longa: A review of preclinical and clinical research. Altern. Med. Rev. J. Clin. Ther..

[28] Borges R.S., Ortiz B.L.S., Pereira A.C.M., Keita H., Carvalho J.C.T. (2018). Rosmarinus officinalis essential oil: A review of its phytochemistry, anti-inflammatory activity, and mechanisms of action involved. J. Ethnopharmacol..

[29] Peterfalvi A., Miko E., Nagy T., Reger B., Simon D., Miseta A., Czéh B., Szereday L. (2019). Much More Than a Pleasant Scent: A Review on Essential Oils Supporting the Immune System. Molecules.

[30] Figueiredo Y.G., Bueno F.C., Júnior A.H.D.O., Mazzinghy A.C.D.C., Mendonça H.D.O.P., de Oliveira A.F., De Melo A.C., Reina L.D.C.B., Augusti R., Melo J.O.F. (2021). Profile of the volatile organic compounds of pink pepper and black pepper. Sci. Electron. Arch..

[31] Silva M.R., Bueno G.H., Araujo R.L.B., Lacerda I.C.A., Freitas L.G., Morais H.A., Augustini R., Melo J.O.F. (2019). Evaluation of the Influence of Extraction Conditions on the Isolation and Identification of Volatile Compounds from Cagaita (*Eugenia dysenterica*) Using HS SPME/GC-MS. J. Braz. Chem. Soc..

[32] Nascimento P.T., Fadini M.A.M., Rocha M.S., Souza C.S.F., Barros B.A., Melo J.O.F., Von Pinho R.G., Valicente F.H. (2021). Olfactory response of Trichogramma pretiosum (Hymenoptera: Trichogrammatidae) to volatiles induced by transgenic maize. Bull. Entomol. Res..

[33] García Y.M., Ramos A.L.C.C., de Oliveira A.H., de Paula A.C.C.F.F., de Melo A.C., Andrino M.A., Silva M.R., Augusti R., de Araújo R.L.B., de Lemos E.E.P. (2021). Physicochemical Characterization and Paper Spray Mass Spectrometry Analysis of *Myrciaria floribunda* (H. West ex Willd.) O. Berg Accessions. Molecules.

[34] García Y.M., de Lemos E.E.P., Augusti R., Melo J.O.F. (2021). Otimização da extração e identificação dos compostos voláteis de Myrciaria floribunda. Rev. Ciência Agronômica.

[35] García Y.M., Ramos A.L.C.C., de Paula A.C.C.F.F., do Nascimento M.H., Augusti R., de Araújo R.L.B., de Lemos E.E.P., Melo J.O.F. (2021). Chemical Physical Characterization and Profile of Fruit Volatile Compounds from Different Accesses of Myrciaria floribunda (H. West Ex Wild.) O. Berg through Polyacrylate Fiber. Molecules.

[36] Mariano A.P.X., Ramos A.L.C.C., de Oliveira Júnior A.H., García Y.M., de Paula A.C.C.F.F., Silva M.R., Augusti R., de Araújo R.L.B., Melo J.O.F. (2022). Optimization of Extraction Conditions and Characterization of Volatile Organic Compounds of *Eugenia klotzschiana* O. Berg Fruit Pulp. Molecules.

[37] Kim S.F., Huri D.A., Snyder S.H. (2005). Inducible Nitric Oxide Synthase Binds, S-Nitrosylates, and Activates Cyclooxygenase-2. Science.

[38] Attiq A., Jalil J., Husain K., Ahmad W. (2018). Raging the War Against Inflammation With Natural Products. Front. Pharmacol..

[39] Zidar N., Odar K., Glavac D., Jerse M., Zupanc T., Stajer D. (2008). Cyclooxygenase in normal human tissues—Is COX-1 really a constitutive isoform, and COX-2 an inducible isoform?. J. Cell. Mol. Med..

[40] Rouzer C.A., Marnett L.J. (2009). Cyclooxygenases: Structural and functional insights. J. Lipid Res..

[41] Moro M.G., Sanchez P.K.V., Lupepsa A.C., Baller E.M., Franco G.C.N. (2017). Biología de la ciclooxigenasa en la función renal–Revisión de la literatura. Rev. Colomb. Nefrol..

[42] Jaradat N., Al-Lahham S., Abualhasan M.N., Bakri A., Zaide H., Hammad J., Hussein F., Issa L., Mousa A., Speih R. (2018). Chemical Constituents, Antioxidant, Cyclooxygenase Inhibitor, and Cytotoxic Activities of Teucrium pruinosum Boiss. Essential Oil. BioMed. Res. Int..

[43] Salaria D., Rolta R., Sharma N., Patel C.N., Ghosh A., Dev K., Sourirajan A., Kumar V. (2021). In Vitro and in silico antioxidant and anti-inflammatory potential of essential oil of *Cymbopogon citratus* (DC.) Stapf. of North-Western Himalaya. J. Biomol. Struct. Dyn.

[44] Al-Maharik N., Jaradat N., Qneibi M., Abualhasan M.N., Emwas N. (2020). Glechoma curviflora Volatile Oil from Palestine: Chemical Composition and Neuroprotective, Antimicrobial, and Cyclooxygenase Inhibitory Activities. Evid.-Based Complement. Altern. Med..

[45] Rollinger J.M., Haupt S., Stuppner H., Langer T. (2004). Combining Ethnopharmacology and Virtual Screening for Lead Structure Discovery: COX-Inhibitors as Application Example. J. Chem. Inf. Comput. Sci..

[46] Batlouni M. (2010). Anti-inflamatórios não esteroides: Efeitos cardiovasculares, cérebro-vasculares e renais. Arq. Bras. Cardiol..

[47] Cerqueira N.F., Yoshida W.B. (2002). Óxido nÌtrico: Revisão. Acta Cir. Bras..

[48] Da Silva C.B., Ceron C.S., Mendes A.S., de Martinis B.S., Castro M.M., Tirapelli C.R. (2021). Inducible nitric oxide synthase (iNOS) mediates ethanol-induced redox imbalance and upregulation of inflammatory cytokines in the kidney. Can. J. Physiol. Pharmacol..

[49] De Oliveira G.A., Cheng R.Y., Ridnour L.A., Basudhar D., Somasundaram V., McVicar D.W., Monteiro H.P., Wink D.A. (2017). Inducible Nitric Oxide Synthase in the Carcinogenesis of Gastrointestinal Cancers. Antioxid. Redox Signal..

[50] Bahadoran Z., Mirmiran P., Ghasemi A., Kashfi K. (2019). Type 2 Diabetes and Cancer: The Nitric Oxide Connection. Crit. Rev. Oncog..

[51] Singh S., Gupta A.K. (2011). Nitric oxide: Role in tumour biology and iNOS/NO-based anticancer therapies. Cancer Chemother. Pharmacol..

[52] Sahebnasagh A., Saghafi F., Negintaji S., Hu T., Shabani-Boroujeni M., Safdari M., Ghaleno H.R., Miao L., Qi Y., Wang M. (2021). Nitric Oxide and Immune Responses in Cancer: Searching for New Therapeutic Strategies. Curr. Med. Chem..

[53] Cinelli M.A., Do H.T., Miley G.P., Silverman R.B. (2019). Inducible nitric oxide synthase: Regulation, structure, and inhibition. Med. Res. Rev..

[54] Özenver N., Efferth T. (2020). Small molecule inhibitors and stimulators of inducible nitric oxide synthase in cancer cells from natural origin (phytochemicals, marine compounds, antibiotics). Biochem. Pharmacol..

[55] Tabanca N., Nalbantsoy A., Kendra P.E., Demirci F., Demirci B. (2020). Chemical Characterization and Biological Activity of the Mastic Gum Essential Oils of Pistacia lentiscus var. chia from Turkey. Molecules.

[56] Prabhakar S.S. (2001). Tetrahydrobiopterin reverses the inhibition of nitric oxide by high glucose in cultured murine mesangial cells. Am. J. Physiol. Physiol..

[57] Maruca A., Moraca F., Rocca R., Molisani F., Alcaro F., Gidaro M.C., Alcaro S., Costa G., Ortuso F. (2017). Chemoinformatic Database Building and in Silico Hit-Identification of Potential Multi-Targeting Bioactive Compounds Extracted from Mushroom Species. Molecules.

[58] Lagunin A.A., Goel R.K., Gawande D.Y., Pahwa P., Gloriozova T.A., Dmitriev A.V., Ivanov S.M., Rudik A.V., Konova V.I., Pogodin P.V. (2014). Chemo- and bioinformatics resources for in silico drug discovery from medicinal plants beyond their traditional use: A critical review. Nat. Prod. Rep..

[59] Salvemini D., Kim S.F., Mollace V. (2013). Reciprocal regulation of the nitric oxide and cyclooxygenase pathway in pathophysiology: Relevance and clinical implications. Am. J. Physiol. Integr. Comp. Physiol..

[60] Maldonado-Rojas W., Olivero-Verbel J. (2012). Food-Related Compounds That Modulate Expression of Inducible Nitric Oxide Synthase May Act as Its Inhibitors. Molecules.

[61] Maia J.G.S., Silva M.H.L., Andrade E.H.A., Zoghbi M.G.B., Carreira L.M.M. (2002). Essential oils from *Astronium urundeuva* (Allemao) Engl. And, A. fraxinifolium Schott ex Spreng. Flavour Fragr. J..

[62] Cole E., Dos Santos R., Lacerda V., Martins J., Greco S., Neto A.C. (2014). Chemical composition of essential oil from ripe fruit of Schinus terebinthifolius Raddi and evaluation of its activity against wild strains of hospital origin. Braz. J. Microbiol..

[63] Elshafie H.S., Ghanney N., Mang S.M., Ferchichi A., Camele I. (2016). An In Vitro attempt for controlling severe phytopathogens and human pathogens using essential oils from mediterranean plants of genus Schinus. J. Med. Food.

[64] Trott O., Olson A.J. (2010). AutoDock Vina: Improving the speed and accuracy of docking with a new scoring function, efficient optimization, and multithreading. J. Comput. Chem..

[65] Zardecki C., Dutta S., Goodsell D.S., Lowe R., Voigt M., Burley S.K. (2022). PDB-101: Educational resources supporting molecular explorations through biology and medicine. Protein Sci..

[66] (2014). The UniProt Consortium UniProt: A hub for protein information. Nucleic Acids Res..

[67] Guex N., Peitsch M.C., Schewde P.T. (2009). Automated comparative protein structure modeling with SWISS-MODEL and Swiss-PdbViewer: A historical perspective. Electrophoresis.

[68] Kim S., Chen J., Cheng T., Gindulyte A., He J., He S., Li Q., Shoemaker B.A., Thiessen P.A., Yu B. (2021). PubChem in 2021: New data content and improved web interfaces. Nucleic Acids Res..

[69] Morris G.M., Huey R., Lindstrom W., Sanner M.F., Belew R.K., Goodsell D.S., Olson A.J. (2009). Olson AutoDock4 and AutoDockTools4: Automated docking with selective receptor flexibility. J. Comput. Chem..

[70] (2010). The PyMOL Molecular Graphics System.

[71] Morandat F., Hill B., Osvald L., Vitek J. (2012). Evaluating the Design of the R Language.

[72] Mweene P., Muzaza G. (2020). Implementation of Interactive Learning Media on Chemical Materials. J. Educ. Verkenn..

[73] Marpaung D.N., Pongkendek J.J., Azzajjad M.F., Sukirno S. (2021). Analysis of Student Motivation using Chemsketch on Hydrocarbon Topic in SMA Negeri 2 Merauke. J. Appl. Sci. Eng. Technol. Educ..

[74] Ragno R. (2019). www.3d-qsar.com: A web portal that brings 3-D QSAR to all electronic devices—The Py-CoMFA web application as tool to build models from pre-aligned datasets. J. Comput. Mol. Des..

[75] Araújo B.M., Coelho A.L., Silveira S.A., Romanelli J.P., de Melo-Minardi R., Silveira C.H. (2019). GAPIN: Grouped and Aligned Protein Interface Networks. bioRxiv.

